# Digitally Planned Reconstruction of a Case of Mandibular Central Giant Cell Granuloma in a Young Female: A Case Report 

**DOI:** 10.30476/dentjods.2026.107038.2713

**Published:** 2026-09-01

**Authors:** Harish Saluja, Anuj Dadhich, Seemit Shah, Shivani Sachdeva

**Affiliations:** 1 Dept. of Oral and Maxillofacial Surgery, Rural Dental College Loni, Maharashtra, India.

**Keywords:** Mandibular reconstruction, Giant cell tumors, Fibula, Esthetic

## Abstract

Reconstruction of maxillofacial continuity defects has invariably been a difficult task for oral and maxillofacial surgeons over the years. Maxillofacial bone defects are caused by the trauma, clefts, burns, and infection, benign, or malignant tumors. To reconstruct big bony defects within the head and neck region remains a serious surgical challenge. The goal of mandibular reconstruction is to revive facial kind and performance, implying repair of mandibular continuity, and muscle attachments. Free-flap reconstruction of oncological mandibular defects has become the fashionable common place of care. Here we are presenting a case of young female having central giant cell granuloma (CGCG) of the mandible in which reconstruction of the defect was done with free fibula flap, which was planned digitally to simulate the anatomy of mandible for better esthetic outcomes.

## Introduction

Facial esthetics has a symbolic importance and is a window of an individual to the world [ 1 ]. The mandibular reconstructions were reported by Hidalgo using the vascularized fibula flaps in the year 1989 [ 2 ]. Free vascularized bone transplant was considered as the gold standard for mandibular defects reconstruction, as it can be harvested with good dimensions and length. Although free flap reconstruction option is a time-consuming and intricate process, it can be justified for improved functional and aesthetic results [ 3 ]. Three-dimensional digital planning of big facial defects have helped surgeon in getting best possible outcome by almost simulating the existing anatomy of the normal bone. The greatest outcomes are achieved by preoperative virtual planning. The availability of cutting guides and prefabricated osteosynthesis plates to maximize conformation, makes surgeon confident and comfortable during procedure, and reduces intraoperative time [ 4 ].

Rapid prototyping and virtual planning offer the several benefits including a way to precisely assess a defect's anatomy, optimal pre-surgical planning, precise preplanning of osteotomies, an optimal graft fit without the need for additional osseous adaptation, reduced surgical time, a highly predictable surgical outcome as reported by Girod *et al* . [ 5 ] and enhanced communication between surgeons and between the surgeon and the patient [ 5 ].

The differential diagnosis of central giant cell granuloma (CGCG) includes other giant cell-containing lesions like brown tumor, aneurysmal bone cyst, cherubism, and odontogenic lesions such as ameloblastoma, odontogenic myxoma, odontogenic fibroma, and non-ossifying fibromas.

Treatment options for CGCG include surgical methods like curettage or en bloc resection for aggressive cases, and non-surgical methods such as calcitonin injections, intralesional corticosteroids [ 6 ]. 

## Case Presentation

**Figure 1:**
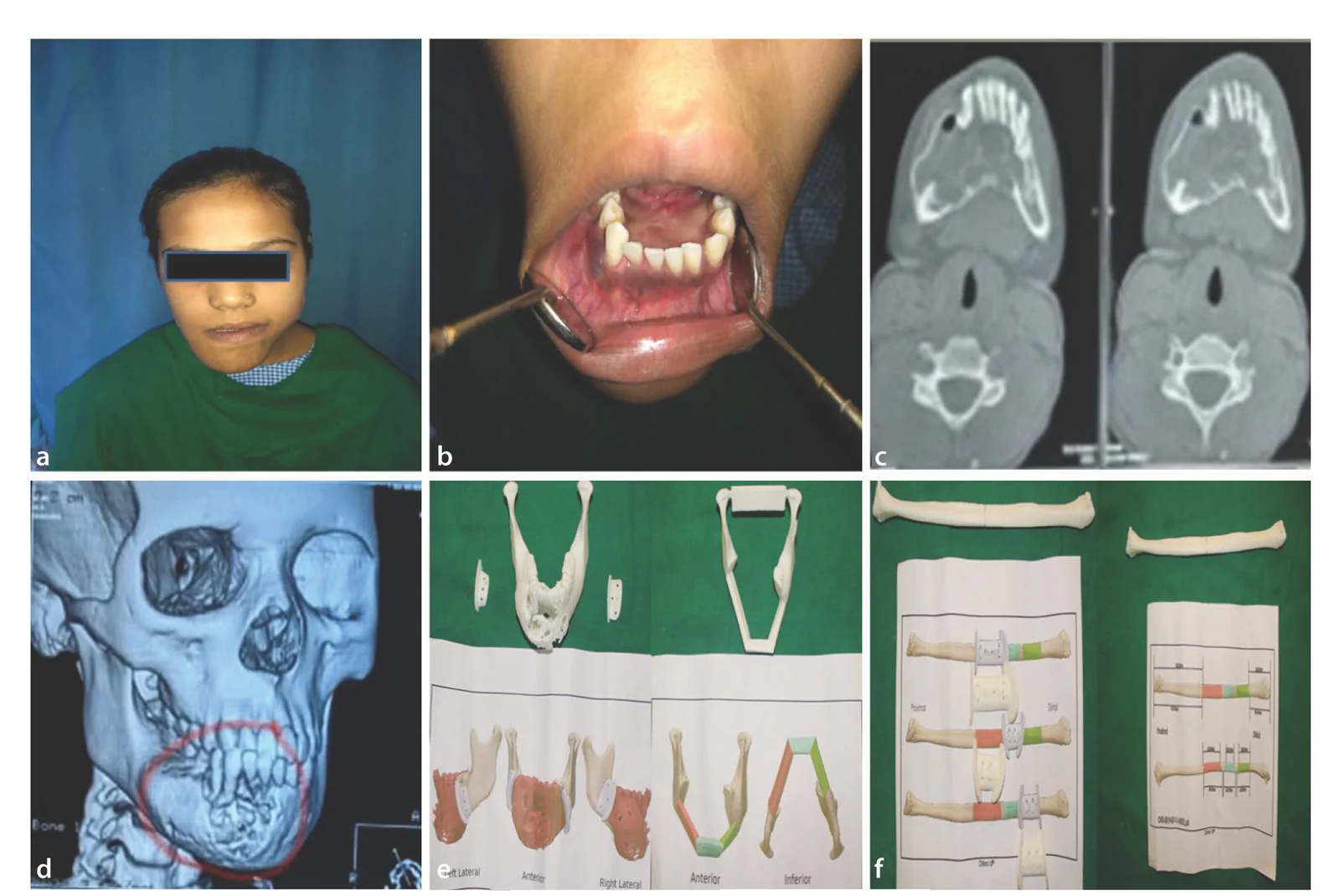
**a:** Showing extra oral swelling over right side of mandible crossing midline, **b:** Intra oral swelling in right lower region with lingual extension, **c:** Preliminary measurements of the resective and reconstructive program from CT scan, **d:** Three-dimensional (3-D) CT reconstruction visualizing the large defect of the right mandible, **e:** A physical 3-D model was designed, which consisted of structurally unchanged elements and parts which needed reconstruction. Resection cuts planned digitally, **f:** Digital models of ﬁbula were obtained and generated utilizing a similar technique. Prepared models were imported to a computer aided design system (CAD)

A 15=year-old female patient reported to the Department of Oral and Maxillofacial Surgery, with the chief complaint of inflammation and swelling over lower half of face since six months. Patient was apparently alright 6 months back when she noticed swelling in anterior mandibular area that initially was smaller in size, gradually increasing, not associated with pain. On extra oral examination, facial asymmetry was present because of a diffuse swelling on lower one third of face on right side (Figure 1a).

Intra oral examination revealed swelling in region of buccal vestibule extending from 43 to 46 regions antero-posteriorly with overlying mucosa stretched with bluish discoloration. In addition, diffuse swelling was noted on lingual aspect. Swelling was non tender and hard in consistency on palpation (Figure 1b). Computer Tomography (CT) scan and incisional biopsy were the investigations advised.

Radiographic investigations showed a large, well defined radiolucency extending antero-posteriorly from 36-47 and super inferiorly from alveolar crest of mandible to low border of mandible with thinning, expansion, and perforation of buccal cortex (Figures 1c-d).

After negative aspiration of the lesion, incisional biopsy of the swelling from right side of mandible was taken. Histopathological report with H &amp; N stained section showed keratinized stratified squamous epithelium. Deeper connective tissue was highly cellular, fibroblastic stroma with proliferative plump, spindle shaped uninuclear mesenchymal cells and collagen fibers showing whorled arrangement along with multinucleated giant cell containing eosinophilic cytoplasm with 5-15 nuclei per cell was seen. Overall histopathological reports were suggesting of CGCG. Even serum calcium, phosphorous, alkaline phosphatase levels, and parathyroid levels were checked where the values of all the tests were within normal limits while only serum calcium levels were little higher than normal limits. In the reported case the lesion was big ,aggressive, with multiple perforations and extension into soft tissue on lingual aspect lead us to opt for aggressive resection approach, but our main aim was focus on delivering best possible esthetic results, so reconstruction was 3D digitally planned. As our case was a big aggressive lesion with perforation in the bone, resection of lesion was planned along with 3D planned reconstruction. 

### Virtual surgical planning

A 3D model of the patient's mandible and fibula was created using computed tomographic (CT) and angiographic CT scans of the patient's lower legs (the donor location) and jaw. Using a handheld Doppler, the lower leg's perforator vessels were located and noted on the skin. Figures 1e-f show the distance measured between the lateral malleolus and the perforator skin vascular. Additionally, the CT scan was used to take initial measures for the restorative and reconstructive procedure.

The 3D acrylic models were prepared of diseases mandible and fibula bone. After exact measurements, model surgery was planned. Exact cutting guides were made for intraoperative purpose to cut bone at required angles. Reconstructed model was prepared according to patient required dimensions.

### Surgery

**Figure 2:**
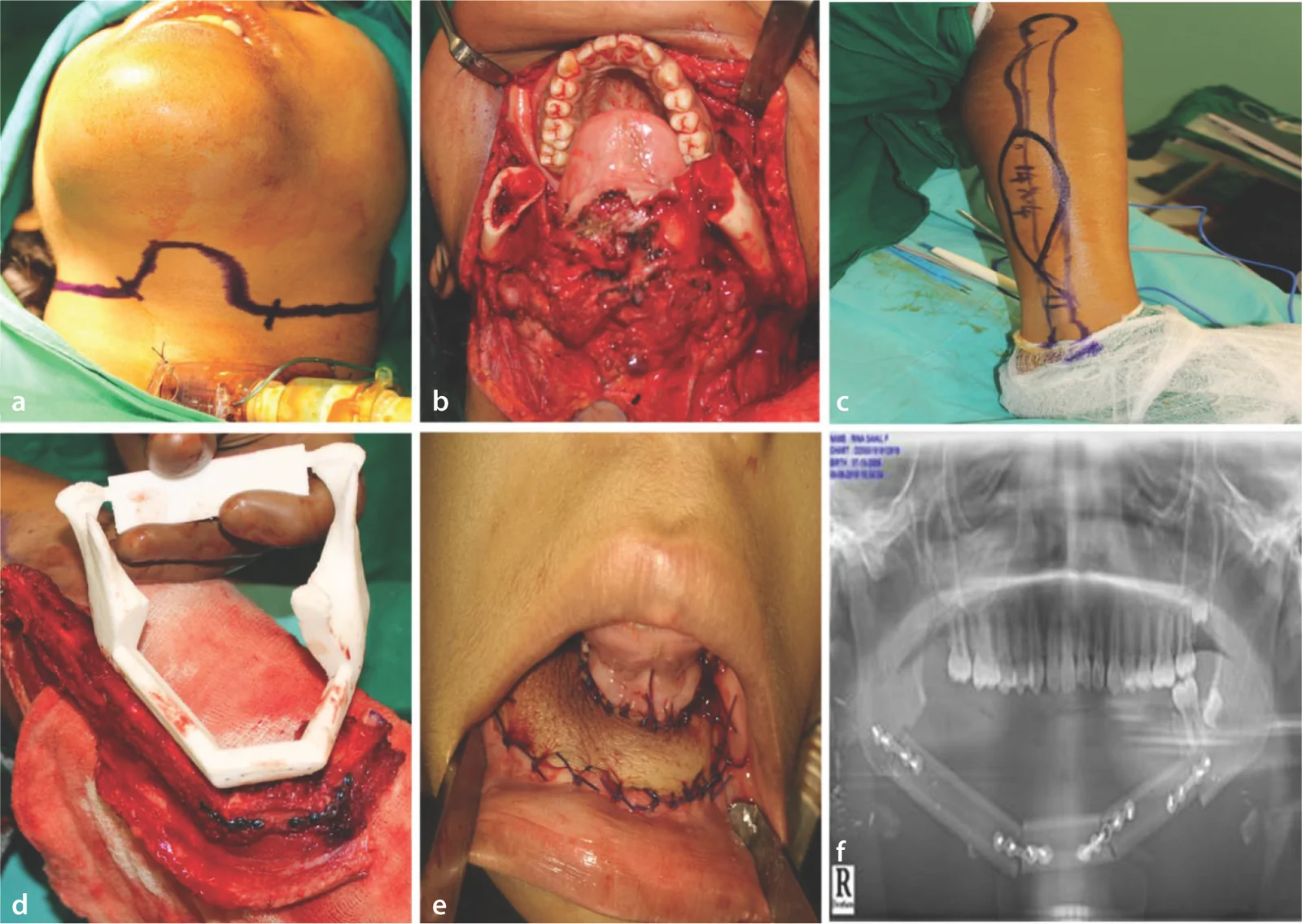
**a:** Incision marking, **b:** Mandibular osteotomies completed with soft tissue resection, **c:** Preoperative measurements of the distance between the malleolus and the perforator vessel, **d:** Shaped fibula secured to the PSP in the planned position and fixation of fibula at surgical defect, **e:** Patient after reconstructive surgery (after anterior segmental mandibulectomy), **f:** Post operative radiographic investigations

Under all aseptic precaution in the operation theatre (OT), patient was induced under general anesthesia. The cutting guides were fastened in the intended location and access to the mandible was created. Trocar guides for pedicle screw placement (PSP) fixation screws and fixation holes for temporary fixation were included in the mandible and fibula cutting guides. The tumor was meticulously excised during the osteotomies, which were performed in accordance with the cutting guidelines. Ultimately, the tumor and soft tissue involved were resected (Figure 2b). Simultaneously, the fibula was harvested by a second surgical team.

Following dissection and perforator vascular identification, the fibular guides were affixed to the bone in order to duplicate the end and closure wedge cuts from previously scheduled osteotomies. The distal and proximal osteotomies of the fibular bones were carried out following the placement and fixing of the cutting guides. A missing piece of fibula reconstructed mandible was re-created. Maintaining appropriate forms and identifying leading curves to achieve the ideal initial shape of the mandible was the most crucial step in the bone filling design process (Figure 2d). 

### Outcome and follow-up

Free fibula graft was successful in maintaining satisfactory esthetic results in the follow up period. Patient is on every 2-month follow up since last 3 years. 

## Discussion

Virtual surgical planning technology is of great use in cases where it requires multiple osteotomies like in big mandibular lesions as mandible is a U shaped bone [ 6 ].

Treatment options for CGCG of jaw bones, includes conservative medicinal therapy like intralesional injections of steroids, interferon and so on to curettage of the lesions and aggressive surgical resections. Abdo EN *et al* . [ 7 ], in their study, treated CGCG of anterior mandible with intralesional steroid. Similar intralesional steroid injection treatment for mandibular giant cell granuloma was performed by Kurtz *et al* . in 2001 [ 8 ]. In our case, the lesion was quite big, aggressive in nature with multiple perforation and lesion extending to lingual soft tissue was the reason behind selection of aggressive approach; similar aggressive treatment was opted by Jeyaraj *et al* . (2019) [ 6 ], Yadav h *et al* . in 2023 [ 9 ] in their reported cases. In our case, expectation of the patient and parents was very high, as patient was young female and was about to get married in coming years. The follow-up results were quite promising with the use of digital planning of the reconstruction procedure and helped us in give patient a good personal and social life. To achieve the highest level of precision in bone restoration, it is crucial to be able to program in the most exact manner at all times. 

## Conclusion

Treatment option for CGCG of jaw bones varies from conservative treatment like intralesional steroids to aggressive options like resection of the pathology depending on the severity of individual case. Digital planning for reconstruction of aggressive cases helps surgeon in delivering better esthetic post-operative results to mimic the preoperative facial appearance. It can be concluded that digital planning should be considered for reconstruction of big facial defects.
